# Editorial Comment: Maximal anatomic bladder neck preservation at the prostatic origin (MANO) in robotic radical prostatectomy: does prostate size matter?

**DOI:** 10.1590/S1677-5538.IBJU.2026.9904

**Published:** 2026-03-10

**Authors:** Luciano A. Favorito

**Affiliations:** 1 Universidade do Estado do Rio de Janeiro Unidade de Pesquisa Urogenital Rio de Janeiro RJ Brasil Unidade de Pesquisa Urogenital - Universidade do Estado do Rio de Janeiro - Uerj, Rio de Janeiro, RJ, Brasil; 2 Hospital Federal da Lagoa Serviço de Urologia Rio de Janeiro RJ Brasil Serviço de Urologia, Hospital Federal da Lagoa, Rio de Janeiro, RJ, Brasil

**Deerush Kannan Sakthivel^1^, Pushan Prabhakar^2^, Mohamed Javid Raja Iyub^2^, Manuel Ozambela Jr^2^, Murugesan Manoharan^2^**

^1^ Department of Urology, Baptist Health South Florida, Miami, USA; ^2^ Department of Urology, Baptist Health South Florida, Miami, USA

**J Robot Surg. 2025 Oct 22;19(1):707.**

DOI: 10.1007/s11701-025-02880-7 | ACCESS: 41125940

## COMMENT

IRobotic surgery has revolutionized the surgical treatment of prostate cancer, resulting in better outcomes and faster recovery ( 1 – 4 ). One of the most important aspects for the technical refinement of this surgical procedure is knowledge of pelvic anatomy. In tis paper of Sakthivel and collegues ( 5 ) we can observe the importance of the anatomy specially in large prostates. The authors described the maximal anatomic bladder neck preservation at the prostatic origin (MANO) technique, designed to enable safe circumferential dissection at the true bladder neck origin irrespective of gland size and concluded that larger prostates were associated with older age and higher preoperative, mean operative time increased with gland size and hospital stay was longer for > 50 cc prostates. The authros shows that the MANO technique enables safe bladder neck preservation across all prostate sizes. Despite increased operative complexity in larger glands, functional and oncological outcomes remain equivalent. This approach may standardize bladder neck management in RALP and support improved continence recovery irrespective of prostate volume. We congratulate the authors for the interesting paper.

## Data Availability

Uninformed

## References

[1] Moschovas MC, Jaber A, Saikali S, Sandri M, Bhat S, Rogers T, Gamal A, Loy D, Patel E, Reddy S, Sighinolfi MC, Rocco B, Harvey T, Ficarra V, Patel V (2024). Impacts on functional and oncological outcomes of Robotic-assisted Radical Prostatectomy 10 years after the US Preventive Service Taskforce recommendations against PSA screening. Int Braz J Urol.

[2] Jiao RD, Wang Z, Kong XG, Tang SY, Xia D, Wu ZJ, Liu JC, Liu LH (2025). Clinical Outcomes and Cost-effectiveness between the Sentire® and da Vinci® systems in Robot-assisted Radical Prostatectomy. Int Braz J Urol.

[3] Gamal A, Moschovas MC, Saikali S, Reddy S, Ozawa Y, Sharma R, Kunta A, Rogers T, Patel V (2025). Comparing the Technological and Intraoperative Performances of Da Vinci xi and DaVinci 5 Robotic Platforms in Patients Undergoing Robotic-Assisted Radical Prostatectomy. Int Braz J Urol.

[4] Pires RDS, Pereira CWA, Favorito LA (2024). Is the learning curve of the urology resident for conventional radical prostatectomy similar to that of staff initiating robot-assisted radical prostatectomy?. Int Braz J Urol.

[5] Sakthivel DK, Prabhakar P, Iyub MJR, Ozambela M, Manoharan M (2025). Maximal anatomic bladder neck preservation at the prostatic origin (MANO) in robotic radical prostatectomy: does prostate size matter?. J Robot Surg.

